# A climate sensitive-health risk: A scoping review of environmental determinants of aquatic injury and mortality

**DOI:** 10.1186/s12889-026-28350-9

**Published:** 2026-07-09

**Authors:** Sean D. Kelly, Robert W. Brander, Jasmin C. Lawes, Amy E. Peden

**Affiliations:** 1 UNSW Beach Safety Research Group, UNSW Sydney, Kensington, NSW 2052 Australia; 2 Surf Life Saving Australia, Bondi Beach, Sydney, NSW 2026 Australia; 3 School of Biological, Earth and Environmental Sciences, Faculty of Science, UNSW Sydney, Kensington, NSW 2052 Australia; 4 School of Population Health, Faculty of Medicine and Health, UNSW Sydney, Kensington, NSW 2052 Australia

**Keywords:** Climate change, Drowning prevention, Injury, Health, Adaptation

## Abstract

**Background:**

Drowning is the third leading cause of unintentional injury death globally. Weather and ocean conditions are recognised as important determinants of drowning and other aquatic injuries (both fatal and non-fatal). Climate change represents an emerging concern increasing aquatic injury risk. This scoping review aimed to identify environmental determinants of unintentional aquatic injury, contextualising the findings within the recognised impacts of climate change.

**Methods:**

Systematic searches across eight databases identified peer-reviewed studies that investigated associations between aquatic injury and weather and ocean conditions. Two reviewers independently screened studies by title/abstract, then full text. Data were extracted using standardised forms, with numerical summary and thematic analysis employed to synthesise findings.

**Results:**

Of 104 included studies, only five directly examined climate change impacts on aquatic injury. Eight environmental determinants were identified as drivers of aquatic injury risk. Strong evidence demonstrated positive exponential relationships between ambient air temperature > 22 °C and aquatic injury risk, with heatwaves representing periods of extreme risk. Humidity, wind speed, cloud cover, and rainfall act as exposure moderators, while wave heights of 0.5–1.5 m, wave periods > 6 s, and low water levels are associated with increased injury risk.

**Discussion:**

Weather and ocean conditions are significant drivers of aquatic injury, constituting a growing climate-sensitive health risk. Climate change will disrupt weather patterns and intensify extreme events, simultaneously increasing human exposure to waterways and water-related hazard intensity. Despite projected increases in climate-driven aquatic injury, there is a paucity of research examining this critical intersection, underscoring an urgent need for targeted investigation and evidence-based adaptation strategies.

**Supplementary Information:**

The online version contains supplementary material available at https://doi.org/10.1186/s12889-026-28350-9.

## Background

The global burden of injury and mortality occurring in aquatic environments – both natural and manmade - is substantial. Drowning is the third leading cause of death resulting from unintentional injury worldwide, with an estimated 300,000 deaths annually [1–3]. In addition to these fatal incidents, a largely unquantified burden of non-fatal drowning also exists [3, 4], characterised by submersion and respiratory impairment from which the individual survives [5]. Beyond drowning, aquatic environments are also associated with a range of other injuries including spinal fractures, blunt force trauma, and lacerations which, at their most severe, can result in lasting physical and neurological impairment, complete incapacitation, or death [6, 7].

Incidents of injury and mortality occurring in aquatic environments – herein collectively referred to as aquatic injury - result from the intersection of exposure to aquatic environments, the degree to which that environment is considered hazardous, the vulnerability of those exposed, and the capability of individuals, populations, and broader health systems to pre-empt, manage and respond to risk [8]. Operating within this framework, weather and ocean conditions can be understood as environmental determinants of aquatic injury [9] – they shape patterns of exposure to aquatic environments and determine how hazardous conditions are at a given time and place [6, 10–12].

Climate change represents an emerging area of concern for the burden of aquatic injury [13, 14], acting as a stress multiplier by significantly altering global temperatures and weather patterns [15–17], as well as the frequency and intensity of extreme weather events [18, 19], in ways that likely influence both exposure to, and the hazard profile of, aquatic environments themselves [13, 18, 20]. The magnitude and regional distribution of these impacts will depend on the degree to which anthropogenic greenhouse gas emissions continue to drive further atmospheric warming [21]. If left unabated, climate change may significantly increase the risk of aquatic injury in the future [13, 14, 20, 22–24], with considerable implications for both the water safety and drowning prevention sector and broader health systems and infrastructure.

Understanding how weather and ocean conditions drive both exposure to, and the formation of, hazardous conditions in aquatic environments, and how climate change will alter these dynamics, is essential for mitigating current and future risks for those recreating in and around the water. While literature describing how weather and ocean conditions act as determinants of aquatic injury risk exists, to date this evidence base has not been systematically characterised. What remains notably sparse are studies describing the impact of climate change on aquatic injury outcomes – either retrospective studies attributing a fraction of the historical burden to experienced changes in climate [25], or prospective studies forecasting future excess injury and mortality under different projected emissions scenarios [8, 26]. Without a clear understanding of these dynamics the water safety sector’s capacity to anticipate future challenges, and direct research and policy efforts accordingly, remains limited.

Scoping review methodologies provide a framework for which evidence existing across a broad range of study designs within a particular research field can be mapped and synthesised in a rigorous manner [27, 28]. Given the often-multidisciplinary nature of water safety research [29], the study design of a scoping review is well suited for this purpose.

This scoping review had two related objectives. First, to identify and describe the ways in which weather and ocean conditions function as determinants of aquatic injury risk. Second, to contextualise these findings within the recognised impacts of climate change, describing the implications of anticipated environmental changes for aquatic injury risk. Where available, attribution and projection studies are examined as a primary source of evidence [8, 25], providing direct estimates of climate change impacts on aquatic injury risk. Recognising that such research remains limited [26], the review then draws on the broader literature, describing anticipated climate-driven changes to weather patterns, ocean conditions, and the frequency and intensity of extreme events. Contextualising the identified environmental determinants of aquatic injury within these projected changes allows for the review to propose plausible pathways through which climate change may alter the distribution and frequency of aquatic injury – establishing aquatic injury as an emerging climate-sensitive health concern and identifying priority areas for future research.

## Methods

This study followed the scoping review methodology described by Arksey and O’Malley [27], incorporating recommendations from Levac et al. [28]. This method was chosen for its iterative process, allowing for the research to be reflexive in its mode of inquiry but still robust in its summation of literature and reporting methodology. The PRISMA for Scoping Reviews reporting tool [30] was used to guide the review reporting structure (S1 file). The protocol for this review was pre-registered on the Open Science Framework Registries (osf.io/wtae5) with transparent changes to the protocol logged in the documentation of the registration.

### Stage 1: Identifying the research question

The original intention of this review was to describe how weather and ocean conditions function as environmental determinants of aquatic injury occurring only in natural water bodies (e.g., oceans, rivers, lakes). However, after piloting a search strategy and reviewing the returned texts it became clear that due to the nature of data generally being obtained at a population level from coronial and hospital records, many relevant studies often did not disaggregate their findings by the water bodies in which the incidents occurred. Further, due to the nature of hydrometeorological disasters leading to land inundation, it is often difficult to discreetly code incidents based on the water body in which they occurred. As such, the scope of this review was expanded to include injuries and fatal incidents occurring across all aquatic environments – freshwater, marine, and man-made. In turn, the research questions explicitly became: (a) how do weather and ocean conditions act as environmental determinants of aquatic injury; and (b) how will the burden of aquatic injury be impacted by climate change? In answering question (b), the review adopts the two-source approach described in the background, first drawing on direct attribution and projection studies and then, where these are limited, on the broader climate change literature to contextualise findings and identify future lines of inquiry.

### Stage 2: Identifying relevant studies

The study aimed to identify published peer-reviewed original works that directly investigated associations between weather and ocean conditions and incidence of aquatic injury. For the purpose of this review, aquatic injuries were defined as drowning and other types of unintentional injury occurring in a body of water, including marine creature attack or envenomation, the outcome of which may be fatal or non-fatal. The study only considered unintentional incidents – the pathways contributing to intentional aquatic injury are beyond the scope of this review. A key methodological decision was made to exclude articles that merely describe potentially hazardous conditions (like rip currents or rough seas) without directly attributing aquatic injuries to these conditions. This approach was chosen to capture behavioural characteristics in the data, based on the hypothesis that people generally make risk assessments and avoid entering water when they perceive conditions pose a significant safety risk [31].

A search string was developed with the aid of an experienced librarian to identify all relevant peer reviewed journal articles from eight different databases (Scopus, Web of Science, Environment Complete, GreenFile, Embase, PubMed, CINAHL, and Informit Full Text). The search strategy was restricted to include only articles published in the English language.

The literature search was conducted on the 13th of January 2025 and considered all relevant literature published prior to this date, with no restriction placed on publication date for inclusion in the study. In brief, the initial search strategy used Boolean search terms to link together three concepts: weather and ocean conditions (inclusive of ambient air and sea surface temperature, wind, storminess, as well as wave and tide characteristics), aquatic injury measures (e.g., drowning, injury), and waterways (beach, river, lake). This search strategy, however, returned an unmanageable number of results (in excess of 15,000), largely unrelated to the research question. The initial planned search was then refined by trialling the removal and addition of terms, with the final search string encompassing three concepts: drowning *AND* weather and ocean conditions; rescue *AND* lifeguard; and surf-zone injury. The justification for the inclusion of the term’s “rescue”, “lifeguard” and “surf-zone injury” is that these terms were identified as being relevant to the literature from a pilot search conducted prior to the development of the search string. The final complete search strategy for each database is available in Supplementary File 2. Studies were then dual screened for inclusion by two of the four authors based on a pre-defined inclusion/exclusion criteria described in stage 3 of the methods below. Citation chaining was then performed on all included studies to complete the search strategy and ensure maximum rigor.

Prior to conducting the formal literature search, a general search of available literature was conducted on Google Scholar and key articles expected to meet the criteria for inclusion in the study were documented. The formal literature search strategy was validated by cross referencing the returned studies with those previously identified in the general literature search and ensuring that all identified relevant articles were returned in the formal search.

### Stage 3: Study selection

The formal search strategy returned a total of 2852 unique studies which were then reviewed for inclusion in the study. Following the recommendations from Levac et al. [28], the study inclusion and exclusion criteria were developed iteratively as familiarity with the research developed over the review process. In piloting the study, the authors first reviewed a sample of 200 articles, then convened to discuss conflicts for inclusion and queries that had arisen from this initial review of the literature. After deliberation, inclusion criteria for the study were defined as follows: (a) be written in English; (b) be a peer-reviewed article presenting new data; (c) report on incidents of fatal or non-fatal aquatic injury among recreational water users or those entering the water unintentionally; (d) report on ten or more incidents (i.e., no case studies); and (e) report a direct association between weather and ocean conditions and the incidence of aquatic injury. Texts were excluded if they: (a) were not human-based studies; (b) were written in a language other than English; (c) did not use new or original data sources (avoiding the duplication of data sources); or (d) centred on intentional water entry as a means of self-harm or incidents occurring in occupational settings – these incidents involve patterns of water use that fundamentally differ from those among recreational water users and require their own distinct and thoughtful examination.

Once these inclusion and exclusion criteria were confirmed, all remaining identified studies were independently screened for inclusion by two members of the research team in two stages, first by title and abstract, then by full text. Screening was conducted using the online software platform Covidence. Lead author SK was the first screener and reviewed all studies. The role of second screener was split between authors AP, RB and JL. The authorship team remained in contact throughout the screening process to discuss challenges and uncertainties in applying the inclusion criteria but remained independent in their decision to either include or exclude a study (Agreement and Cohen’s Kappa for title and abstract and full text screening available in S3 file). For cases where two authors conflicted on their decision, a third author was consulted to resolve the conflict.

### Stage 4: Data extraction

Data extraction was performed by lead author SK using a standardised form encompassing five sections: study information, exposure characteristics, population characteristics, study methods, and epidemiological burden and outcome measures. The data extraction form was first piloted on ten studies, with results examined by the research team to review accuracy and completeness of the data extracted. The form was then modified to improve useability and applied to data extraction for all included studies. The final data extraction form is included as Supplementary File 4.

### Stage 5: Collating, summarising, and reporting the results

Methods of numerical summary and thematic analysis were used to summarise the extracted data [27, 28]. Data were reported in the form of a narrative summary for each environmental determinant identified. The number of studies relating to each environmental determinant was reported to describe the relative depth of evidence across determinants and highlight areas where further research is required.

Studies were also characterised and summarised according to where the study was performed (the study country of origin), the burden of aquatic injury in that country in accordance with Institute for Health Metrics and Evaluation Global Burden of Disease Study data on drowning [32], and the degree of vulnerability of the country to the effects of climate change in accordance with the European Commission Joint Research Centre Index for Risk Management (INFORM) [33]. This allowed for study findings to be contextualised within broader environmental and health vulnerability frameworks, identifying research gaps and enabling assessment of whether research efforts are appropriately distributed relative to global drowning burden and climate-related risk.

## Results

**Fig. 1:**
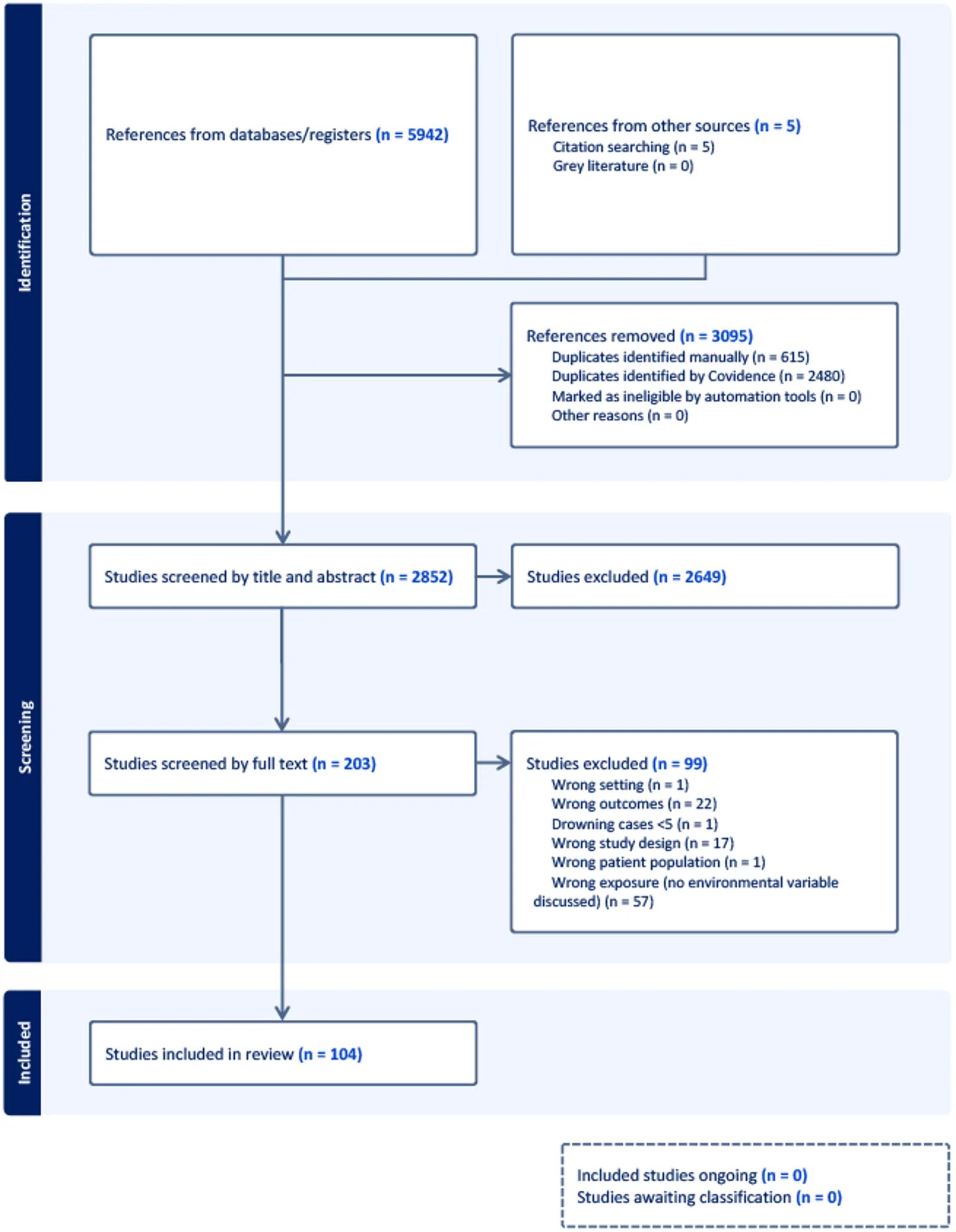
Modified PRISMA-ScR flow chart

A total of 2,852 unique studies were identified by the search strategy (2,847 from database searches and five from citation chaining), of which 104 met criteria for inclusion following title, abstract, and full-text screening (Fig. 1, S5 file).

### Study characteristics

**Fig. 2:**
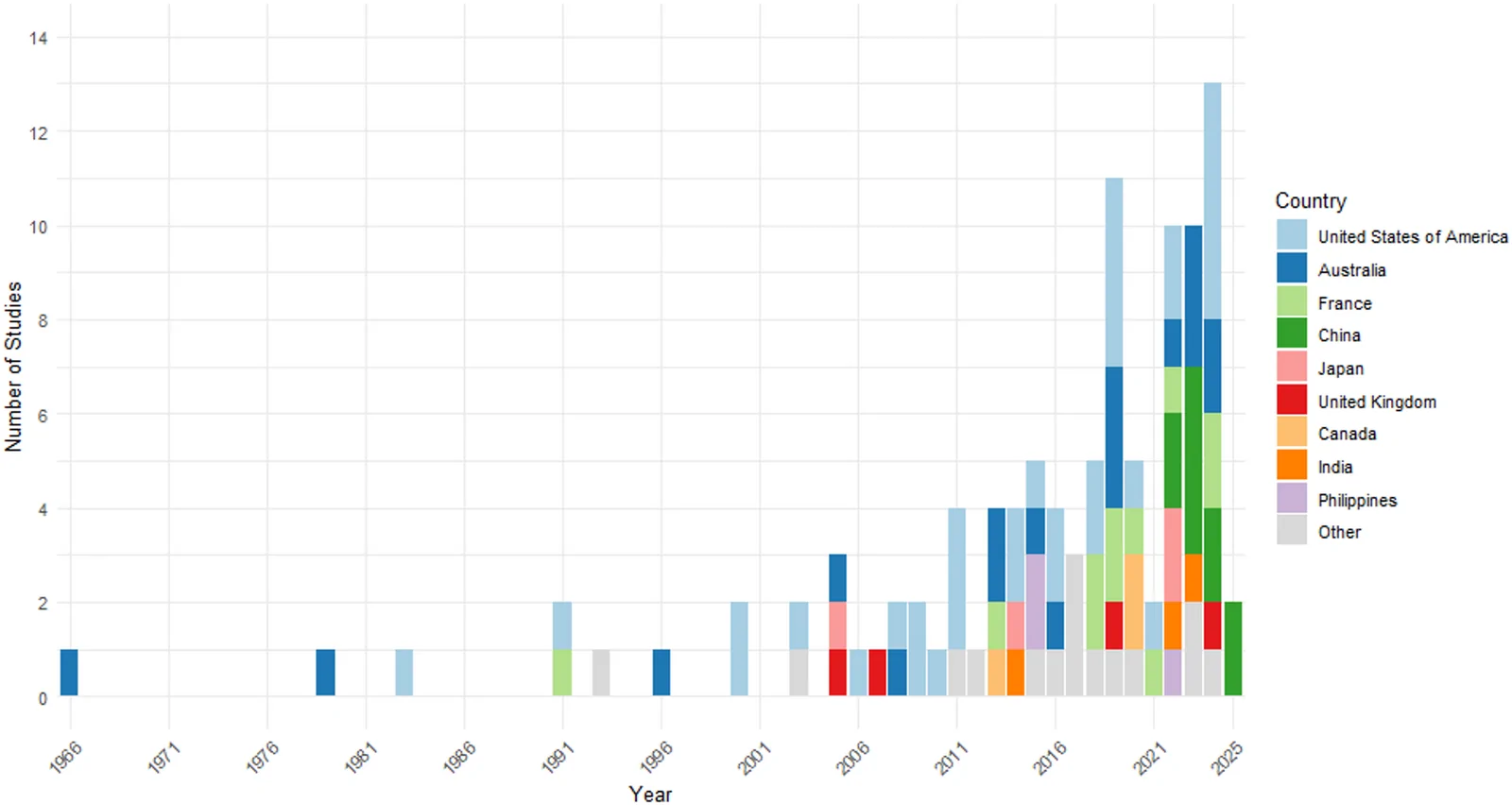
Included studies (*n* = 104) by country (*n* = 20) and publication year

**Fig. 3:**
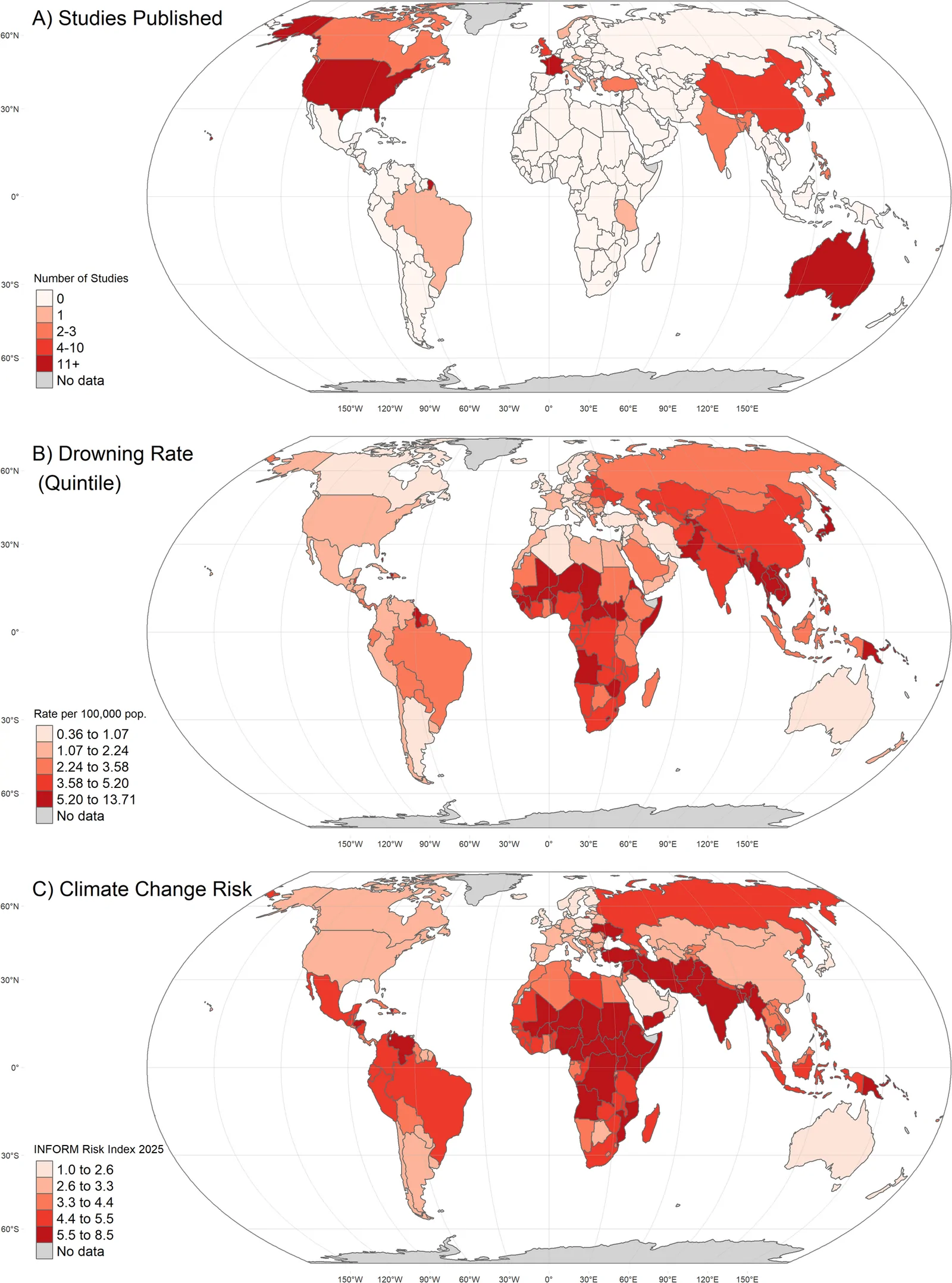
Global distribution of aquatic injury research, fatal drowning burden, and climate risk. Panel **A**: Number of aquatic injury studies by country. Panel **B**: Drowning mortality rates per 100,000 population (quintiles) based on the Global Burden of Disease study on drowning (2021) [32]. Panel **C**: Vulnerability to climate-related hazards as presented by the INFORM Climate Risk Index 2025 [33]. Note: Small island states excluded due to scale

Few studies were published prior to 2004 (10.7%, *n* = 11), with the earliest published in 1966 from Australia (Fig. 2). After 2004, there was a progressive exponential increase in publications. Collectively, three-quarters of studies comprised data from only six countries (77.9%, *n* = 81), 34 (32.7%) from the United States of America, 18 (17.3%) from Australia, 11 (10.6%) from France, ten (9.6%) from China, and four (3.8%) from both Japan and the United Kingdom, with the remaining 23 (22.1%) coming from 14 other countries (Fig. 3). Only 11 (10.6%) studies originated from countries classified as high-risk for climate change effects under the INFORM Risk Classification 2025 (Fig. 3) [33]. The majority came from lower-risk nations: 37 (35.6%) from medium-risk countries, 44 (42.3%) from low-risk countries, and 12 (11.5%) from very low-risk countries. The distribution of studies showed a clear inverse relationship with drowning rates (Fig. 3) with only seven (6.7%) originating from countries with the highest drowning rates (top quintile globally) [32], while nearly three-quarters came from countries with the lowest drowning rates (bottom two quintiles: 48 (46.2%) from the fourth quintile and 29 (27.9%) from the lowest quintile). The remaining studies came from countries with moderate drowning rates: 17 (16.3%) from the second-highest quintile and 3 (2.9%) from the middle quintile.

### Epidemiological characteristics

When examining the data types presented in the studies, 58.7% (*n* = 61) analysed fatality data exclusively, 10.6% (*n* = 11) focused solely on non-fatal data, and 30.8% (*n* = 32) examined both fatal and non-fatal outcomes. Drowning was the most studied injury mechanism, appearing in 77.9% (*n* = 81) of all studies. Other injury mechanisms were investigated in 58.7% (*n* = 61) of studies, with sprains, fractures, and blunt force trauma appearing most frequently among these alternatives (31.7%, *n* = 33). The data sources used in the included studies were generally of a high degree of reliability, most commonly drawn from coronial data/death certificates (50.0%, *n* = 52), often supplemented by hospital admission (15.4%, *n* = 16), emergency service (34.6%, *n* = 36), and beach lifesaving personnel data (20.2%, *n* = 21). Only in 6 studies were lower levels of evidence solely used (6%), namely media reports and newspaper articles.

A diversity of analytical approaches was used across included studies to examine the relationship between environmental determinants and aquatic injury outcomes (supplementary file 5). Measures of effect (including risk and odds ratios) calculated from inferential statistical models were reported in 42.3% (*n* = 44) of studies. These inferential studies included regression models, case-crossover designs, and machine learning methods. Other studies reported descriptive statistics, including measures of central tendency and proportion of incidents occurring under given environmental conditions (57.7%, *n* = 60) – this was common among studies relating to natural hazards, likely due to difficulties with attribution and reliability of data collection following these events. This methodological diversity creates an interpretive challenge, as drawing conclusions across studies using different designs, data sources and outcomes limits direct comparison. These methodological differences are considered further in the context of the review’s limitations.

### Climate change characteristics

Only five studies (4.8%) conducted original analyses on the impacts of climate change on aquatic injury, all in relation to ambient air temperature (S5 file). Of the remaining 99 studies, 21 (20.2%) discussed climate change impacts without performing original analyses on projected aquatic injury, while the majority (78 studies;75.0%) did not consider or made no statement regarding the impacts of climate change. Among the studies that did consider the impact of climate change, all uniformly found that aquatic injury is highly sensitive to rising temperatures, and that drowning is likely to be one of the injury mechanisms most affected by climate change.

### Environmental characteristics

The review identified eight environmental determinants of aquatic injury risk (Table 1), each representing a discrete ambient weather or ocean condition. The literature also surfaced a distinct category of natural hazard events – including tropical cyclones, floods, and rip currents – which, while themselves not weather and ocean conditions, represent emergent features of the review, describing the compounding consequences of the underlying environmental conditions and the projected impacts of climate change. These were narratively synthesised separately, as their inclusion reflects the scope of the discovered literature rather than a primary focus of the review. The most frequently studied environmental determinants of aquatic injury risk were ambient air temperature and ocean wave characteristics (including height, period, and direction), each examined in 32 publications (30.8%), followed by tidal characteristics including water level, tidal phase, and spring-neap tide cycle (17.5%, *n* = 18). A total of 47 studies considered more than one environmental determinant in their analysis.

**Table 1:** Number of studies by environmental determinant reported and whether study considered climate change in analysis and findings

	Environmental Determinant	Number of Studies (%)	Consideration of Climate Change in Study		
			Conducted Original Analysis	Discussed, but No Original Analysis Performed	Not Considered in Study
Ambient Weather and Ocean Conditions	Air temperature	32 (30.8%)	5 (15.6%)	10 (31.3%)	17 (53.1%)
	Wave characteristics	32 (30.8%)	0 (0%)	2 (6.3%)	30 (93.8%)
	Tidal characteristics	21 (20.2%)	0 (0%)	1 (4.8%)	20 (95.2%)
	Wind	20 (19.2%)	0 (0%)	3 (15%)	17 (85%)
	Rainfall	13 (12.5%)	0 (0%)	5 (38.5%)	8 (61.5%)
	Cloud cover	8 (7.7%)	0 (0%)	1 (12.5%)	7 (87.5%)
	Water temperature	6 (5.8%)	0 (0%)	0 (0%)	6 (100%)
	Humidity	5 (4.8%)	0 (0%)	3 (60%)	0.4 (0%)
Natural Hazards	Rip current	26 (25.0%)	0 (0%)	1 (3.8%)	25 (96.2%)
	Tropical cyclone	22 (21.2%)	0 (0%)	5 (22.7%)	17 (77.3%)
	Flood	15 (14.4%)	0 (0%)	8 (53.3%)	7 (46.7%)

Associations and reported measures of effect between environmental determinants and aquatic injury risk are summarised in Table 2 and natural hazard impacts qualitatively summarised in Table 3, with supporting information provided in Supplementary File 5. There is a strong consensus among studies that a positive exponential relationship exists between ambient air temperature and aquatic injury risk at temperatures exceeding 22 °C (17/18 relevant studies; 94% agreement), with heatwave conditions representing periods of extreme risk (5/5 relevant studies; 100% agreement). Moderate evidence exists showing that in colder climates, drowning risk is highest when temperatures approach 0 °C, and lowest when temperatures drop below − 5 °C (3/3 relevant studies; 100% agreement). There is modest evidence suggesting associations between sea surface temperature (SST) and aquatic injury risk, with risk generally higher during periods when SST exceeds 19.5 °C (2/2 relevant studies;100% agreement).

**Table 2:** Summary of reported association and measures of effect between environmental determinants and aquatic injury and mortality risk among those who enter the water voluntarily

Environmental Determinant	Overall Association with Aquatic Injury	Agreement (*n*; Citations)	Disagreement (*n*; Citations)	Reported Measures of Effect
Ambient Air Temperature				
Heatwave/Extreme heat	Increased risk during heatwaves/compound heat extremes.	5 [10, 24, 34–36]	0	RR = 1.09–1.26. OR = 3.02 (95% CI: 2.39–3.83). Attributable fraction: 2.8%-11.4%.
Air temperature > 10–22 °C	Positive exponential relationship with rising temperature.	17 [23, 24, 35, 37–50]	1 [51]	Risk increases 2.06–7.2% per 1 °C increase in mean daily temperature. Odds 1.1-12.2x higher > 22 °C vs. 10–22 °C.
Effect moderated by humidity	Positive relationship moderated by relative humidity.	1 [47]	0	Excess risk higher on dry-hot days: 32.34% (95% CI: 24.64–40.50) vs. wet-hot days: 14.38% (95% CI: 6.80–22.50).
Warm/Hot days	Higher frequency on warm/hot days (> 20 °C).	8 [43, 52–58]	0	Aquatic injuries frequently overrepresented on days > 20 °C.
Air temperature < 10–15 °C	Inverse relationship, peaking at 0 °C.	2 [41, 42]	0	RR drowning death 2.33x higher at 0 °C vs. 22 °C; OR at 0 °C = 1.05 vs. 15 °C.
Negative temps (-5 °C to 0 °C)	Risk increases exponentially as temp approaches 0 °C.	1 [59]	0	Winter air temperature explained 48% of variation in winter drownings across countries. Fixed effects model: Winter drowning incidents = 1.5 + 9.3e^0.4t^ *p*(<0.001).
Impact on survival	No significant association with survival when submerged (only mild to warm climates examined).	2 [60, 61]	0	Survival odds stable (adj. OR = 1.042, *p* = 0.361). Minimal temp difference between survivors/non-survivors.
Humidity				
Overall injury risk	Generally higher on days with low relative humidity.	3 [35, 42, 47]	1 [51]	Lower humidity levels associated with higher odds of aquatic injury (OR = 1.78 for < 69.1% relative humidity vs. >78.8% relative humidity). Dry-hot ER = 32.34% vs. wet-hot ER = 14.38%. One study found no significant association (< 60% RH).
Impact on survival	Worse on days with high relative humidity.	1 [60]	0	Fatal cases had significantly higher humidity levels (71.2%) compared to survivors (57.5%, *p* < 0.001).
Sea Surface Temperature (SST)				
SST > 19.5˚C	Positive relationship with increasing temperature, with higher frequency on high sea surface temp days.	2 [37, 44]	0	OR = 3.95 (19.5–21.3 °C); OR 13.18 (> 21.3 °C) vs. <19.5 °C. High SST (> 24.3 °C) associated with increased surf zone injury risk.
Survival correlation	Rates of survival positively correlated with SST.	1 [62]	0	Significant negative correlation with fatalities (b=-0.0526, *p* < 0.001). Fatalities are linked to water temperatures ~ 1 °C cooler than those for survivors.
Marine creature encounter	Significant association with marine creature encounter.	2 [63, 64]	1 [65]	Envenomation’s most frequent between 18.8˚C-19.5˚C. Shark attacks at mean 25.5˚C (23-28.7˚C). Cooling waters correlated with shark bites.
Wind Speed				
Surf zone injury	Highest on days with low wind speeds (2–7 m/s).	10 [37, 38, 43, 52–54, 56, 57, 66, 67]	2 [55, 68]	> 85% injuries on light winds (< 7 m/s). Injuries repeatedly overrepresented on low wind. Conflicting: one study no relationship and one shows inverse relationship.
Boating fatality	Higher during periods of high wind.	2 [69, 70]	0	Odds of fatality increased 90% (OR = 1.9, 95% CI: 1.0-3.6). 56% drownings under strong wind/heavy rain.
Rip current injuries	Highest at wind speeds <5 m/s.	2 [66, 67]	1 [68]	50%-100% incidents <5 m/s. One study: 90% drownings > 5.14 m/s.
Wind Direction				
Surf zone injury	Not related to overall numbers but impacts specific hazards.	3 [54, 68, 71]	1 [55]	Conflicting evidence. Offshore winds more common with rescues (32.7% vs. 16.8%). Onshore winds more common with no rescues (67.5% vs. 52.3%). One study: 64.2% rescues during onshore winds.
Rip current injuries	Highest during periods of onshore winds.	2 [68, 71]	0	70%-100% fatalities associated with onshore winds.
Cloud Cover				
Overall risk	Likelihood lower on days with high cloud cover.	5 [37, 44, 52, 57, 72]	3 [48, 51, 73]	Injury likelihood reduced OR = 0.52 (95% CI: 0.37–0.74). Higher rates with solar radiation > 740 W/m2. High beach attendance on clear days. Conflicting: some no relationship; one study higher risk with high cloud (OR = 5.7, poor visibility).
Rainfall				
Recreational water users	Higher likelihood of injury on days with no rainfall.	5 [35, 42, 43, 48, 56]	3 [45, 51, 70]	Slightly increased injury days with no rainfall (RR = 1.17). Odds of drowning decrease 1.3%-4.5% per 1 mm rainfall. Positive association for fishers. Conflicting: some studies show no relationship. Higher rainfall positively associated with increased likelihood of aquatic injury among fishers (land and boat-based).
High rainfall periods	Increased risk of injury, primarily due to flooding.	3 [42, 46, 74]	0	U-shape relationship with drowning risk, lowest at 16.6 mm. Higher risk > 180.55 mm. Positive association (EV = 0.291–0.315 per mm).
Wave Height				
Surf zone injury	Significant association, inverted-U relationship (0.5–1.5 m).	11 [6, 53, 54, 56, 57, 67, 72, 75–78]	4 [38, 79–81]	Rescues show positive relationship for wave heights < = 4ft (IRR 3.25) and negative relationship for wave heights >4ft (IRR). Highest rates 0.5–1.5 m. Highest rates/proportion of injury repeatedly shown to fall between ~ 0.5 to 1.5 m.
Rip current injury	More frequent at larger wave heights (0.5–2.25 m) with evidence of regional variation.	6 [38, 67, 79–82]	1 [78]	80% Pacific rip drownings: 0.75–1.75 m. 70% Caribbean: 1.25–2.25 m.
Wave Period				
Surf zone injury	Moderate to long periods (6–12 s) generally increased risk.	8 [37, 53, 54, 57, 67, 75, 79, 83]	1 [56]	Highest rates 6–12 s. One study: 4–5 s.
Rip current injury	Impact varies significantly by location/morphology.	3 [67, 79, 81]	0	Caribbean: 6.5–9.5 s. Pacific: 12.5–14.5 s. India: 5–8 s. US: 6–7 s.
Wave Direction				
Surf zone injury	Highest when near shore normal.	4 [53, 56, 57, 67]	0	Accounts for up to 70% of injuries.
Rip current injury	Highest when near shore normal.	1 [67]	0	Accounts for 70% of rip current drowning deaths.
Water Level				
Surf zone injury	Highest during low tide/low water level.	7 [6, 54, 68, 77, 79, 84, 85]	5 [31, 38, 53, 61]	Injury risk 40% lower at other times (RR = 0.6). 8% reduction per hr away from low tide (IRR = 0.92). Conflicting evidence: 36.8%-75% at low water; some studies indicate greater risk at high tide, site-specific variation, or no association.
Rip current injury	Highest during low water levels.	3 [38, 68, 79]	0	Predominantly occurred at low tide (75% within 2 h prior/4 hrs after).
Tide Phase (ebb/flood)				
Surf zone injury	Highest during ebb tide phase.	3 [54, 77, 79]	2 [85, 86]	59.4% injuries during ebb. One study: board rider risk higher during flood.
Spring-Neap Tide Cycle				
Surf zone injury	Highest during spring tide.	5 [38, 53, 67, 82, 87]	2 [56, 57]	Incidents increase 2 days before & 3 days after new moon. Linked to larger tidal ranges. Conflicting evidence: higher during neap tide.
Rip current injury	Highest during spring tide.	3 [38, 67, 82]	0	Higher frequency recorded during spring tide (larger tidal ranges).

Abbreviations: *RR* rate ratio, *OR* odds ratio, *ER* excess risk, *IRR* incident rate ratio, *aOR* adjusted odds ratio

**Table 3:** Studies reporting risk factors for aquatic injury and mortality associated with natural hazards and unintentional water entry

Category	Risk Factor	Overall Association with Aquatic Injury	Agreement (*n*; Citations)	Disagreement (*n*; Citations)	Reported Measures of Effect
Tropical Cyclone (TC)/Storm Surge					
Demographic	Age	Older population (> 55 years) and children (< 10 years) represent high risk demographic for TC injury.	11 [88–99]	2 [100, 101]	> 55 years: OR = 3.0 (95% CI: 1.7–6.0). highest mortality rate seen in those aged 80+ (9.74/1 million pop). ~2x deaths among those over 45. IRR for > 65 years = 1.227 (95% CI: 0.955–1.576). Frequently overrepresented comprising between 26.8% and 49% of fatal incidents. The median age of those who fatally drowned higher than those who survived (49 years vs. 30 years). <10 years: IRR for < 5 years = 1.294 (95% CI: 0.985-1.700). frequently overrepresented comprising between 4.8% to 26% of incidents.
	Sex	Males are frequently overrepresented for TC injury.	12 [89–91, 93, 95, 97, 98, 100–104]	2 [96, 105]	Mortality rate 1.87 to 1.9x higher. Frequently overrepresented comprising between 54.8% and 77% of incidents.
	Remoteness	Mortality risk associated with TC higher in regional/rural areas.	2 [89, 101]	0	TC mortality risk shown to decrease as urbanisation increases IRR = 0.843; 95% CI: 0.696–1.021. Metropolitan mortality rate = 0.55–0.94 deaths per 1 million pop vs. regional mortality risk 3.23–6.19 deaths per 1 million pop.
Geographic	Proximity to coastline	Mortality risk higher among those living closer to the coast ( ~ < 400 m).	6 [89, 92, 94, 96, 98, 101]	0	Those living within 50 m of the coast OR = 3.9 (95% CI: 2.1–7.3). Lower mortality risk farther from coast IRR = 0.689 (95% CI: 0.604–0.785).
	Low-lying areas	Mortality risk higher among those living in low lying areas.	1 [94]	0	Houses built in low-lying areas with only one story shown to not provide adequate means for people to seek refuge and take higher ground.
Environmental	Wind speed	Positive relationship between wind speed and mortality risk.	2 [89, 106]	0	A one-knot increase in wind speed was associated with a 4% increase in the total number of fatalities. For each 1 standard deviation increase in wind speed, the mortality rate from typhoons increases by 243% (IRR 3.434, 95% CI: 2.969–3.972).
	Rainfall volume	Positive relationship between rainfall volume and mortality risk.	1 [106]	0	A one-inch increase in rainfall was associated with a 28% increase in the total number of fatalities.
Behavioural	Swimming ability	Mortality risk higher among those who have are unable to swim.	1 [92]	0	OR = 3.5 (95% CI: 1.9–6.5).
	Failure to evacuate	Mortality risk higher among those who fail to evacuate or seek appropriate shelter/disregard for early warning messaging/poor communication and community understanding of risk.	6 [88, 92, 94, 95, 104, 107]	0	aOR = 10 (95% CI: 3.8–29.1). Eary warning signs ignored in 22% of deaths.
	Inadequate/Poor housing quality	Mortality risk higher in poor quality and low-rise housing.	3 [88, 90, 107]	0	Deaths more frequently occurred in smaller buildings (86% low rise or 1–4-unit apartments), commonly in basements, and in housing made of weaker/less resilient materials.
	Being outdoors during the storm	Mortality risk higher among those who exited the refuge area during the storm.	2 [92, 101]	0	aOR = 3.6 (95% CI = 1.9–7.1).
	Driving during the storm/through flooded area	High proportion of fatalities commonly occurring among those who drive during the TC.	3 [99, 103, 108]	0	Stated to account for between 46% and 76% in studies that investigated driving as a risk factor.
Flood					
Demographic	Age	Older population (> 65 years) represent high risk demographic for flood mortality.	7 [98, 99, 101, 105, 109–111]	2 [112, 113]	~ 60 to 73% of fatalities. Drowning rate among those aged > 70 years 44.6% higher than the expected.
	Sex	Males are frequently overrepresented for flood injury.	7 [98, 104, 105, 111–114]	0	~ 58.5% to 68.2% of fatalities.
	Remoteness	Mortality risk associated with Floods higher in regional/rural areas.	3 [101, 111, 114]	0	RR = 1.17 to 56.71 (relative to those living in major cities).
	Disability	Those with physical disabilities/existing health issues at higher risk of injury.	1 [109]	0	Among those who had access to a second story floor in their home but did not evacuate upstairs and subsequently drowned, 60% of those had pre-existing disabilities, limiting their ability to evacuate upstairs.
Geographic	Living in flood prone areas	Those living in previously designated flood prone areas at increased risk.	1 [109]	0	Those who lived in areas designated as high-risk flood prone areas on flood maps were at the greatest risk.
Environmental	Water depth	Positive relationship between floodwater depth and mortality risk.	1 [98]	0	Mortality risk was significantly higher in areas where water depth exceeded 2.5 m.
	Flow velocity	Positive relationship between flow velocity and mortality risk.	1 [98]	0	Areas with high depth-velocity (> 5 m²/s) products experienced greater impacts to infrastructure and higher rates of mortality. Generally, areas with extensive building damage saw higher mortality rates.
	ENSO phase	Higher risk of mortality during El Nino ENSO phase.	1 [115]	0	Death toll from floods 220% higher during El Nino years.
Behavioural	Inadequate/Poor housing quality	Mortality risk higher in poor quality and low-rise housing.	2 [109, 110]	0	33% of those who drowned in their home lived in a single-story house, with no option to evacuate to higher ground.
	Failure to evacuate	Mortality risk higher among those who fail to evacuate or seek appropriate shelter/disregard for early warning messaging/poor communication and community understanding of risk.	3 [104, 110, 116]	0	Lack of an effective early warning system noted reduce preparedness and impact survival outcomes. Inadequate evacuation warnings commonly associated with high casualty events.
	Driving through flooded area	High proportion of fatalities commonly occurring among those who drive through a flooded area.	4 [99, 105, 111, 116]	0	Stated to account for between 39.7% and 43% in studies that investigated driving as a risk factor.

Abbreviations: *RR* rate ratio, *OR* odds ratio, *ER* excess risk, *IRR* incident rate ratio, *aOR* adjusted odds ratio

The weight of evidence suggests that humidity, wind speed, cloud cover, and rainfall act as moderators of recreational water use and exposure to aquatic environments, with water use lower on days with high humidity, high wind speed, high cloud cover and rainfall. Evidence also suggests that wave and tide conditions in ocean environments are significantly associated with aquatic injury risk, with the greatest risk occurring at waves heights of 0.5–1.5 m (17/22 relevant studies; 77% agreement), wave periods > 6 s (8/9 relevant studies; 89% agreement), shore-normal wave incidence (5/5 relevant studies; 100% agreement), and during periods of low water levels (3/5 relevant studies; 71% agreement) and greater tidal ranges (8/10 relevant studies; 80% agreement).

Regarding the impacts of severe weather events and associated natural hazards (Table 3), injury and mortality most commonly occur among children and elderly populations, males, and those with physical limitations or disabilities. Poor quality housing and infrastructure, an inability to seek appropriate shelter, and delayed response times are often associated with higher injury and mortality rates, with outcomes often worse among those who have had repeated exposure to events, due to depletion of community resources and response infrastructure. While those living in low lying coastal zones are often seen to be at higher risk, largely due to the impacts of storm surge, it should be noted that those living inland are also at substantial risk of tropical cyclone related injury or death as freshwater rivers and creeks bulge and overflow.

## Discussion

This scoping review demonstrates that weather and ocean conditions are significant determinants of aquatic injury risk. Projected climate change will substantially alter these conditions, likely shifting the burden of aquatic injury - positioning it as an emerging climate-sensitive health risk that warrants greater research attention and targeted prevention efforts. From across the 104 studies included in this review, eight environmental determinants were found to have previously been identified as drivers of aquatic injury risk. While not explicitly the primary focus of the review, natural hazards – including tropical cyclones, floods, and rip currents – were also identified as emergent consequences of these underlying determinants, and as such, are discussed in that context to highlight the compounding impacts of climate change on aquatic injury.

These determinants can be understood to function in two conceptually distinct ways: some drive exposure by shaping when and how people engage in water recreation or come into contact with water unintentionally, while others affect the degree of danger present in aquatic environments by modifying the inherent risks associated with aquatic activities at particular times and places. Importantly, weather and ocean conditions do not act independently of one another, but function in concert, with the greatest risk occurring when periods of high hazardousness coincide with periods of high exposure and water use [37, 38, 52–55].

It is unequivocal that the oceans, lands, and the atmosphere have warmed as a result of climate change [15]. These changes, along with associated changes in weather patterns and extreme climate events have direct implications for aquatic injury risk as environmental conditions continue to change into the future. Here the findings of the review are synthesised and discussed in relation to the existing evidence on climate change, with implications for prevention and future research considered.

### Available evidence base discussing climate impacts on aquatic injury risk

Only a small number of studies (*n* = 5) were identified as having directly examined the impact of climate change on aquatic injury, though the available evidence consistently indicates that aquatic injury risk is highly sensitive to rising temperatures, with projected increases in exposure to hazardous conditions likely to substantially increase the aquatic injury burden in the coming decades [23, 24, 34, 39, 40]. Given the scale of the projected impacts and the considerable geographic variability in how the effects of climate change will be experienced, there is a substantial need to expand this research to inform risk assessment, adaptation planning, and preparedness efforts.

Of particular concern is the geographic overlap between countries currently experiencing the highest aquatic injury burden and those projected to face the greatest climate change impacts. These countries – predominantly low- and middle-income countries (LMICs) [117] – typically have the least capacity for climate adaptation and injury prevention [13, 33]. This concern is compounded by the geographic skew of the existing research base, with studies predominantly conducted in high income countries, leaving the populations at greatest risk the least represented in the evidence base. Climate change therefore risks deepening existing global inequities in the burden of aquatic injuries. Addressing this issue requires not only expanded research in these settings, but deliberate investment in climate adaptation and injury prevention capacity in the regions of greatest need.

### Ambient temperatures

The relationship between aquatic injuries and ambient temperature follows a U-shaped curve, with injury risk greatest at both high and low temperatures and lowest at moderate temperatures [41, 42]. The evidence linking aquatic injury risk and higher ambient temperatures is substantial. Research has repeatedly shown that as temperatures rise above ~ 20 °C to 22 °C, the risk of drowning significantly increases [35, 40, 41, 43–46, 53, 54], with rates of increase as high as 7.2% for every 1 °C increase in mean daily temperature [45]. This association is primarily attributable to increased recreation in aquatic environments and higher alcohol consumption on hot days leading to riskier behaviours around waterways [36, 40, 45, 55], with young males typically identified as the highest risk demographic [39, 40, 45]. This effect may be exaggerated during periods of heatwave and compound heat extremes, where temperatures remain high for multiple days throughout the day and night [10, 24, 34, 36]. However, this effect is likely moderated by humidity, with the risk of drowning on wet-hot days lower when compared to dry-hot days [42, 47].

Evidence linking increased drowning risk during periods of extreme cold (< 10–15 °C) is scarce, however notable associations have been found in studies from colder climates [41, 42]. While drowning incidents are less frequent during periods of extreme cold, those that do occur tend to result in higher fatality rates, with mortality risk inversely correlated with air temperature [41, 59]. The primary mechanism attributed to this increase in risk is poor thermoregulation in cold waters, which can decrease the time before hypothermia takes effect in the water and exacerbate pre-existing medical conditions [41], ultimately, decreasing survival time.

This effect appears to be age-dependent, with older males considered the most vulnerable cohort [41], while younger age groups may actually experience decreased risk at extremely low temperatures [35]. This age disparity likely stems from two factors: relatively higher rates of aquatic recreation among older age groups during colder periods compared to younger age groups, and the protective effect of supervision typically provided to younger individuals. Interestingly, Sharma et al. [59] demonstrated that as temperatures fall below − 5 °C, drowning risk decreases significantly across all demographics due to ice formation on natural water bodies, which substantially reduces direct contact with flowing water.

These temperature-related risks should be understood as being region specific, with different hazard profiles across cold and hot climates. However, the impacts of climate change and global heating are expected to universally increase aquatic injury risk [23, 24, 34, 39, 40, 59]. In warmer regions, rising annual mean temperatures will likely elevate aquatic injury risk as people spend more time in and around water [23, 24, 34, 39, 40]. Similarly, in colder climates, previously ice-covered water bodies will remain unfrozen for longer periods annually, also leading to greater water exposure [59], and therefore increased aquatic injury risk.

Notably, multiple studies indicate that as temperatures rise, drowning mortality attributable to ambient temperature is expected to increase more substantially than any other category of unintentional injury-related death [23, 24, 34, 39, 40]. In China, a country with large climate variability across its landmass, He et al. [23] found that relative to 2010, excess accidental drowning deaths are expected to increase by ~ 10% under 1.5˚C of warming, ~ 28% under 2˚C of warming, and ~ 45% under 3˚C of warming. These findings underscore the urgent need for targeted aquatic injury prevention strategies now and in the coming decades, as rising temperatures cause changes in individual behaviour and temporal patterns of recreation in aquatic locations. In response to these anticipated changes, aquatic education in the form of swimming lessons and risk awareness training [118–120] will become increasingly important into the future, alongside the strategic expansion of lifesaving personnel service provision in public swimming areas [118].

### Sea surface temperatures

Few studies have directly explored the association between sea surface temperature and aquatic injury. Sea surface temperature can be highly correlated with ambient air temperature and, as such, is often excluded as a variable of significance in modelling studies of environmental determinants of aquatic injury [44]. However, it has been shown to uniquely impact the risk profile of aquatic recreation, with concerning implications as oceans warm as a result of climate change [15, 121].

Sea surface temperatures have been positively associated with survival outcomes among those in need of rescue in the water, with survival rates higher for those in warmer waters [62]. While reasonable to conclude that survival outcomes may improve as oceans warm, these benefits will likely be offset by associated increases in aquatic recreation (and subsequent exposure to aquatic injury risk) due to warmer ambient air and ocean temperatures [37].

Further, sea surface temperature directly impacts the behaviour and migration patterns of various marine creatures, including certain shark and ray species, with significant variations across creatures and species [63–65]. When these patterns intersect with patterns of water recreation among humans, the risk of injury from marine creature attack or envenomation can increase [63, 64]. Ocean warming, by altering the temporal and geographic distribution, migration, and abundance of marine species like sharks and rays [122–124], consequently has the potential to shift the rates and distributions of human-wildlife encounters and conflicts [123], however the real world implications of this are not yet well understood.

### Wind, cloud cover and rainfall

Wind, cloud cover and rainfall collectively can influence rates of aquatic injury by impacting risk exposure, acting as incentives or deterrents for people participating in outdoor water activities. Rates of aquatic injury have repeatedly been shown to be higher on days of low cloud cover [37, 51, 52, 73], no rainfall [35, 43, 48], and low wind speed [37, 43, 52, 53, 56, 66], with people more likely to swim and participate in water activities on sunny calm days. Conversely, days of moderate to high cloud cover, rainfall, and wind tend to reduce drowning risk by deterring people from entering the water.

Climate change is expected to shift rainfall patterns as rising temperatures increase the atmosphere’s capacity to hold moisture, while also causing associated changes in atmospheric circulation [125, 126]. These shifts are projected to result in increases in both the frequency and intensity of extreme rainfall events [125, 127], and, in some areas, longer dry periods with less rainfall [125, 126]. These impacts are unlikely to be uniform, as regional differences in projected rainfall trajectories will mean that the net effect on aquatic injury risk will vary considerably by location and time of year. In regions where climate change produces longer dry spells, aquatic recreation and associated injury risk may increase, particularly when these dry periods intersect with warmer months that are themselves peak periods for aquatic recreation. Conversely, in regions projected to experience increased rainfall frequency, deterrence effects may reduce drowning risk – though episodic extreme events could produce distinct hazard profiles of their own, including dangerous surf conditions, flooding and storm surge. Given these complex interactions and uncertainties, targeted research examining the relationship between changing precipitation patterns and aquatic injury rates across different geographic and seasonal contexts is essential for developing evidence-based injury prevention strategies and informing public health preparedness in a changing climate.

Projections for cloud cover and wind speed are considerably more uncertain than those for temperature and precipitation. While the Intergovernmental Panel on Climate Change (IPCC) state with a medium to high level of confidence that cloud height and lateral distribution will shift, the effects on cloud thickness and type, as well as the moderating role of clouds and atmospheric conditions on solar radiation, remains less understood [19, 128]. Changes in mean wind speeds are projected to be relatively modest [19, 129, 130], however, generally poorer model performance currently makes meaningful day-to-day inference difficult [19]. Given these uncertainties, estimates of the influence of projected changes in cloud cover and wind speed on aquatic injury risk are currently constrained. Importantly, the influence of wind on aquatic injury can be further considered in relation to its impact on ocean wave dynamics, particularly during periods of extreme weather.

### Wave characteristics and interactions with beach state, tidal characteristics, rip current formation

Wave characteristics are significantly associated with aquatic injuries in surf zones, representing a complex hazard for beachgoers, lifesaving personnel and coastal risk managers. Independently, wave height significantly affects behaviour patterns of beachgoers, directly influencing individual decisions about water entry. The relationship between wave height and aquatic injuries follows an inverted U-shaped relationship, with most injuries occurring when waves measure between 0.5 and 1.5 m [6, 31, 37, 53, 54, 72, 75]. Wave heights below 0.5 m carry less energy and are more manageable for those in the water, resulting in fewer injuries [53, 54, 72]. Conversely, waves exceeding 1.5 m in height act as a deterrent for people to enter the water altogether, reducing exposure and overall injury rates [53, 72].

Critically, when people do enter the water during high wave conditions, any resulting injuries tend to be more severe, with significantly higher mortality risk [79–81]. This may be most apparent during periods of extreme weather events, such as tropical cyclones and storm surge, which have the potential to generate large swells and wave heights [102]. These conditions are particularly hazardous for anyone in the water, including surfers who often seek out these extreme conditions at great risk to their own safety [102]. Boaters are also at higher risk under these conditions as large swells and rough seas have the capacity to overflow decks and capsize smaller vessels [69, 76].

Wave characteristics including height, period and direction also interact with, or are modulated by, environmental conditions such as tidal state and beach and surf zone morphology, to influence hazards and risk profiles for water users. Rip currents are recognised as the leading hazard on surf beaches [11, 84, 131, 132]. Research shows that rip current rescues and drowning deaths occur more frequently during specific conditions: larger wave heights [38, 52], longer wave periods [52, 79], and nearshore normal wave direction, where waves approach perpendicular to shore [38, 57, 68]. These conditions align with hydrodynamic studies showing increased rip current flow intensity under these circumstances [52, 133]. Tidal state also significantly influences rip current behaviour, with many studies documenting increased rates of injury and mortality during periods of lower tide [38, 67, 68, 79]. This may be explained by interactions between tidal state and beach morphology influencing wave breaking intensity that drives rip current behaviour, creating variable spatial and temporal patterns of rip current risk [52, 80, 133].

Shore breaking waves represent another significant hazard for beachgoers, associated with increased risk of spinal injury [6, 134]. Similar to rip currents, risk increases with larger waves, longer wave periods, and near shore normal wave direction [6, 52, 53, 75, 134]. Shore break injuries typically occur during higher water levels [38, 52, 53] and on steeper beach profiles [37, 134], where waves break closer to shore with more forceful plunging characteristics. However, this relationship varies by location, with some studies finding contradictory evidence of increased risk during low tide, suggesting complex interactions between tidal conditions and local beach morphology [6, 31, 75].

Climate change is expected to impact wave climates via increased intensity of storm events and higher water levels, leading to episodic increases in wave height and energy [135, 136]. Changes in atmospheric pressure systems, storminess, and a poleward shift of tropical cyclone activity may also intermittently increase wave heights in some regions [19, 135, 137–139], although predicted changes in modal wave conditions are modest, with greater uncertainty than projections for other climate variables [140, 141].

Importantly, the hazard posed by these climate-driven changes to wave conditions will be modulated by tidal characteristics, including water level, ebb/flood phase, and spring-neap tide cycles. While tides themselves represent a fixed phenomenon that will not be impacted by climate change, the intersection of high tide periods with episodes of increased storm intensity and associated elevated wave heights represent extremely hazardous conditions for those who enter the water [142, 143]. Consequently, tidal characteristics can be understood as an effect moderator to the impacts of climate change on the coastal wave hazard and the associated impact on aquatic injury.

Changes in beach morphology are also expected as sea levels rise and increase coastal inundation, leading to long-term shoreline retreat and potentially the complete disappearance of some beaches as the sea advances towards fixed land and infrastructure [135, 138, 144]. Changes in wave climate and increased storm intensity will further contribute to more severe coastal erosion and altered patterns of sediment transport, impacting the shoreface and beach alignment [135, 138, 144].

These changes in wave climate and coastal conditions may make shorelines episodically or persistently more dangerous, increasing risks for beachgoers, while their combined effect may also undermine individuals’ familiarity with local hazards at beaches they frequently visit, leaving them more vulnerable to emergent dangers. However, predicting future aquatic injury rates requires accounting for human behaviour as a critical moderating factor. People typically adjust their activities by avoiding water entry during visibly hazardous conditions, potentially offsetting some increased risk. While uncertainty remains about how changing wave climates will ultimately impact rates of aquatic injury, these environmental and behavioural factors represent significant determinants of present-day aquatic injury risk and should be incorporated into real-time prediction models to better inform beach safety protocols and public warnings.

### Severe and extreme weather events and their impacts on unintentional water entry

Severe and extreme weather events have the capacity to cause serious injury and loss of life, along with lasting damage to infrastructure and natural environments. At their most extreme, these events can result in hundreds of thousands of lives lost [88]. A significant proportion of deaths from these events result from drowning [89, 90, 107, 109, 112, 116], often in combination with blunt force trauma as people are swept away in flowing waters and come into contact with floating debris [91].

Tropical cyclones represent a particularly significant threat. These events cause aquatic injury primarily through two main mechanisms: first, through low pressure systems generating powerful winds, which in turn drive storm surge and coastal inundation; and second [142, 145], through heavy rainfall causing subsequent flooding [145]. Climate change is likely to exacerbate these effects as higher baseline sea levels will mean that storm surges will penetrate further inland and cause more extensive flooding compared to equivalent storms under previous sea level conditions [146–149]. Additionally, climate change is likely to exacerbate the compound effect of rainfall-induced flooding during tropical cyclones [145]. Projected increased cyclone intensity, and seawater evaporation [145, 150], as well as the increased capacity of a warmer atmosphere to hold greater moisture content [151, 152], will likely produce more extreme rainfall during severe tropical cyclone events, ultimately increasing flood risk and rates of aquatic injury.

Climate change is projected to increase both the severity and impacts of extreme storm events [17–19]. While the total frequency of some storm types is projected to remain stable or slightly reduce [18], climate models project shifts towards a greater proportion of higher-intensity storms (category 4–5) [18], substantially increasing the risk of severe flooding and storm surge events [146, 147, 149], though regional variations are expected [18]. It has been estimated that under high emission scenarios, approximately 40% of the globe will experience a doubling in the frequency of flood events by 2050 [153]. As these events become more common, human exposure to hazardous aquatic environments will inevitably increase. These impacts will likely be most pronounced in low- and middle-income countries, where the proportion of individuals living in flood-prone areas is higher, and where community infrastructure standards and disaster literacy and preparedness are generally poorer [13].

### Policy and practice implications

Climate change will place increasing pressures on public health and water safety authorities, not only through intensifying hazards, but by reshaping when, where, and how people interact with aquatic environments. These pressures will not be felt uniformly, as vulnerability and adaptive capacity varies substantially across and within nations, making region specific, evidence-based response essential. Foundational to this is the continued investment in high-resolution regional climate modelling – these guide effective climate policy and better inform adaptation efforts [8, 26, 154]. In order for these models to be effective in their purpose, their outputs must be accessible and sensitive to the needs of the agencies that rely on them. As such, ensuring multi-sectoral engagement in model development is critical, with public health, water safety, and environmental monitoring agencies involved early enough to shape what is produced, making outputs usable to the agencies they intend to serve [154]. This integration across sectors is essential to translating climate projections into region specific climate resilience.

To mitigate the worst individual and community impacts, public health and water safety authorities will need to adopt a broad range of adaptation efforts involving risk evaluation, communication, proactive action and response. Improvements to water safety education are essential, including learn to swim programs, water rescue skills, and safety procedures during extreme weather events [88, 92, 93, 107, 155]. It is also necessary to improve the climate resilience of current infrastructure, including housing and protective structures such as sea walls and flood barriers designed to withstand intensifying tropical cyclones [90, 94, 156]. Further to this, the development of more accurate and accessible flood mapping methods and data sets are essential to identify high-risk areas and enable informed decision-making by residents, planners, and policymakers [109]. These actions themselves are dependent on the evidence base described above, as without reliable monitoring of environments and regional climate projections, the ability for agencies to act is constrained. Critically, these actions are resource intensive, and any adaptation effort is ultimately dependent on a community’s capacity to act on the information available to them. Equitable implementation will require substantial financial investment and global cooperation, with targeted support directed towards communities most vulnerable to climate-driven aquatic injury risk.

### Strengths and limitations

Research on aquatic injury spans many disciplines. This study presents a comprehensive review and synthesis of literature specifically examining how weather and ocean conditions drive aquatic injury risk. A key strength of the approach was the inclusion of only those studies that demonstrated direct links between environmental conditions and aquatic injury outcomes. This methodology allowed for identification of both how environmental conditions directly contribute to aquatic injuries while also capturing characteristics of human behaviour and decision making regarding how people interact with potentially hazardous environments.

This work, however, should be considered with several important limitations. While comprehensive, it is possible that all relevant texts were not captured in the search strategy. Data sources reported on in studies were generally of high quality, with the majority of included studies reporting on data obtained from coronial, hospital, or emergency service data. However, inconsistent drowning definitions across jurisdictions along with the likely underreporting of non-fatal injuries is a known limitation in research attempting to quantify and describe the burden of aquatic injury [4].

Heterogeneity in methodological design limited direct comparison of effect estimates across studies. Further, as a significant portion (57%) of included studies described the distribution of incidents across environmental conditions rather than formally estimating measures of effect, the strength of conclusions that could be drawn were constrained. Included studies also often lacked the statistical power to formally examine how environmental determinants affected specific demographic subgroups. Future research employing inferential statistical designs – including longitudinal, time-series, and machine learning approaches – would strengthen attribution of environmental determinants to aquatic injury outcomes and better enable examination of how these relationships vary across different demographic and geographic contexts.

The review’s focus on weather and ocean conditions as determinants of aquatic injury, combined with the relative scarcity of literature explicitly examining environmental determinants of injury in inland and man-made settings, means that the majority of studies discussed centre on coastal and ocean environments. Where inland and man-made environments have been considered in the literature, injury data are frequently aggregated across water bodies, rather than examined in isolation. As a result, while environmental determinants across all aquatic settings are discussed, greater attention is paid to coastal and ocean environments. Further research explicitly examining environmental determinants of aquatic injury in inland and man-made water bodies Is warranted.

Additionally, while the impacts of natural hazards including tropical cyclones, floods, and rip currents were discussed, these emerged as consequences of the underlying weather and ocean conditions being investigated rather than as a primary area of inquiry within the research itself. A comprehensive examination of natural hazards as determinants of aquatic injury and of the projected impacts of climate change on their frequency and severity would require a dedicated review.

## Conclusion

Weather and ocean conditions and associated natural hazards are significant determinants of aquatic injury. Climate change will disrupt established weather and ocean conditions, and intensify extreme meteorological events, simultaneously altering human behaviours that drive exposure to water, as well as increasing the intensity and associated danger of water-related hazards. The most pronounced effects are likely to be driven by increased aquatic recreation due to higher ambient temperatures as well as increases in the intensity and severity of extreme weather events. Importantly, these changes will not be experienced equally, but rather will be region-specific, with some areas more adversely affected than others.

Avoiding the worst of these anticipated impacts will depend on global efforts to reduce emissions and limit global heating, as well as adaptation efforts of nation states and individuals acting to ensure their own safety and preparedness. Water safety education and promotion will become increasingly important as global temperatures and sea levels rise. Substantial benefits can be achieved through sustained, collaborative and cross-sectoral efforts to improve aquatic injury prevention strategies, particularly when targeted at vulnerable populations. Despite the anticipated significant increase in aquatic injury burden due to climate change, research examining this critical intersection remains notably absent from the literature. There is, therefore, an urgent need for future research to focus on the relationship between climate change and aquatic injury risk, to both amplify its importance across the water safety sector, and to inform evidence-based adaptation and prevention strategies.

## Supplementary Information

Supplementary Material 1

Supplementary Material 2

Supplementary Material 3

Supplementary Material 4

Supplementary Material 5

## Data Availability

The authors have made every attempt to be transparent in their data extraction and analysis, providing detailed referencing of sources included in text tables and supplementary material. All data collected during this review are made freely available upon reasonable request.
