# Supplementary material for: A climate sensitive-health risk: A scoping review of environmental determinants of aquatic injury and mortality
Source: BMC Public Health. 2026 Jul 9;26:2752. doi: 10.1186/s12889-026-28350-9 (PMC13632288; doi:10.1186/s12889-026-28350-9)
Supplement: Supplementary file 1 [file 12889_2026_28350_MOESM2_ESM.pdf]

Database Search Strategies

Database search run:13/01/2025

EMBASE (OVID)

1. drown*.mp. [mp=title, abstract, heading word, drug trade name, original title, device manufacturer, drug manufacturer, device trade name, keyword heading word, floating subheading word, candidate term word]
2. (heat* or hot or tide or tidal* or wave* or wind* or weather* or storm* or exposure* or "rip current" or surf or forecast*).mp. [mp=title, abstract, heading word, drug trade name, original title, device manufacturer, drug manufacturer, device trade name, keyword heading word, floating subheading word, candidate term word]
3. 1 and 2
4. (rescue* adj5 (lifeguard* or lifesaver)).mp. [mp=title, abstract, heading word, drug trade name, original title, device manufacturer, drug manufacturer, device trade name, keyword heading word, floating subheading word, candidate term word]
5. ("surf zone injur*" or "surf-zone injur*").mp. [mp=title, abstract, heading word, drug trade name, original title, device manufacturer, drug manufacturer, device trade name, keyword heading word, floating subheading word, candidate term word]
6. 3 or 4 or 5
7. limit 6 to (Human and English language)
8. Filters applied (Publication type = Article)

Articles returned (n): 715

Scopus

1. (TITLE-ABS-KEY (drown*) AND TITLE-ABS-KEY (heat* OR hot OR tide OR tidal* OR wave* OR wind* OR weather* OR storm* OR exposure* OR "rip current" OR surf OR forecast*)) AND (LIMIT-TO (SRCTYPE , "j")) AND (LIMIT-TO (DOCTYPE , "ar")) AND (LIMIT-TO (LANGUAGE , "English"))
2. TITLE-ABS-KEY (rescue* W/5 (lifeguard* OR lifesaver*)) AND (LIMIT-TO (SRCTYPE , "j")) AND (LIMIT-TO (DOCTYPE , "ar")) AND (LIMIT-TO (LANGUAGE , "English"))
3. TITLE-ABS-KEY ("surf zone injur*" OR "surf-zone injur*") AND (LIMIT-TO (DOCTYPE , "ar")) AND (LIMIT-TO (LANGUAGE , "English"))

Articles returned (n): 2,078

PubMed (Medline)

1. (drown*[tiab]) AND (heat* OR hot OR tide OR tidal* OR wave* OR wind* OR weather* OR storm* OR exposure* OR "rip current" OR surf OR forecast* [tiab])
2. "surf zone injur*" OR "surf-zone injur*" [tiab]
3. "rescue lifeguard"[tiab:~5] OR "rescue lifesaver"[tiab:~5]
4. Limiters [Article Language = English; Species = Human)

Articles returned (n): 525

Web of Science

1. Drown* (Topic) and heat* OR hot OR tide OR tidal* OR wave* OR wind* OR weather* OR storm* OR exposure* OR "rip current" OR surf OR forecast* (Topic) [Refined By: Document Types = Article; Languages = English]
2. "surf zone injur*" OR "surf-zone injur*" (Topic) [Refined By: Document Types = Article; Languages = English]
3. rescue* NEAR/5 lifeguard* (Topic) or rescue* NEAR/5 lifeguard* (Topic) [Refined By: Document Types = Article; Languages = English]

Articles returned (n): 1,734

CINAHL / Environment Complete / Greenfile (ebsco)

1. drown* AND (heat* OR hot OR tide OR tidal* OR wave* OR wind* OR weather* OR storm* OR exposure* OR "rip current" OR surf OR forecast*) [Limiters: Language = English; Source Types = Academic journals]
2. "surf zone injur*" OR "surf-zone injur*" [Limiters: Language = English; Source Types = Academic journals]
3. rescue* AND (lifeguard* OR lifeguard*) [Limiters: Language = English; Source Types = Academic journals]

Articles returned (n): 847

Informit Full text collections (Aust.)

1. drown* AND [heat* OR hot OR tide OR tidal* OR wave* OR wind* OR weather* OR storm* OR exposure* OR "rip current" OR surf OR forecast*] AND Limit To: Peer Reviewed AND Resource Type: Journal AND Language: English

2. "surf zone injur*" OR "surf-zone injur*" AND Limit To: Peer Reviewed AND Resource Type: Journal AND Language: English
3. rescue* AND lifeguard* OR lifesaver* AND Limit To: Peer Reviewed AND Language: English AND Resource Type: Journal

Articles returned (n): 38
