# Supplementary material for: A climate sensitive-health risk: A scoping review of environmental determinants of aquatic injury and mortality
Source: BMC Public Health. 2026 Jul 9;26:2752. doi: 10.1186/s12889-026-28350-9 (PMC13632288; doi:10.1186/s12889-026-28350-9)
Supplement: Supplementary file 2 [file 12889_2026_28350_MOESM4_ESM.pdf]

Scoping Review Data Extraction

* Required

* This form will record your name, please fill your name.

Study Information

1. First Author Last Name *

2. Year Published *

3. Full title of paper *

4. Journal *

5. Volume *

6. Issue *

7. Starting page *

8. DOI *

9. Abstract *

10. Country of study *

11. Study period - first year *

12. Study period - end year *

13. Aim of the study *

Exposure Characteristics

14. Body of water/location category *

- [ ] Beach
- [ ] Offshore
- [ ] Bay
- [ ] River/Creek/Estuary
- [ ] Lake/Dam
- [ ] Floodwaters
- [ ] Non-specific
- [ ] Other

15. Weather conditions/Natural hazards (check all that apply) *

- [ ] Air temperature
- [ ] Water temperature
- [ ] Wave characteristics (height, period, direction)
- [ ] Tidal period
- [ ] Cloud cover
- [ ] Rainfall
- [ ] Rip current
- [ ] Flood
- [ ] Hurricane/Tropical cyclone/Typhoon/Storm surge
- [ ] Wind
- [ ] Other

Population

16. Age group studied *

- ◯ Children and adolescents (under the age of 18)
- ◯ Adults only (age 18 years and over)
- ◯ All ages/non-specific

17. Total population based or specific population *

- ◯ Total population
- ◯ Other

18. Study geographic catchment area (e.g., country, state, region) *

19. Activity participating in at time of water entry (check all that apply) *

- ☐ Swimming/Wading
- ☐ Non-powered watercraft use
- ☐ Boating
- ☐ Unintentional water entry
- ☐ Non-specific
- ☐ Other

Methods

20. Study design *

21. Data sources for the study (check all that apply) *

- [ ] Coronial data/Death certificates
- [ ] Hospital admission data
- [ ] Emergency service data
- [ ] Beach lifeguard/lifesaver rescue or first aid data
- [ ] Newspaper/Media reports
- [ ] Other

22. Data analysis methods used (check all that apply) *

- [ ] Univariate or Bivariate
- [ ] Multivariable - covariates identified a priori
- [ ] Multivariable - exploratory/no specific hypothesis or question
- [ ] Other

23. Summary of applied statistical methods (i.e., regression, ANOVA) *

24. Study sample size *

Epidemiological Burden and Outcome Measures

25. this paper includes data on *

- ◯ Drowning only
- ◯ Non-drowning related aquatic injury only
- ◯ Drowning and other aquatic injury
- ◯ Rescue incidents
- ◯ Other

26. Specifically, what type of incidents were reported on *

- ☐ Drowning
- ☐ Spinal
- ☐ Sprain/Fracture/Blunt force trauma
- ☐ Laceration/abrasion
- ☐ Marine creature attack/envenomation
- ☐ Neurological
- ☐ Cardiac
- ☐ Exposure/Hypothermia
- ☐ No specific type of incidents reported (genera grouped fatal/non-fatal injuries with no cause specified)
- ☐ Other

27. Proportion of incidents attributed to drowning *

28. Proportion of incidents attributed to other causes of injury *

29. What outcomes were reported in the study (check all that apply) *

- [ ] Fatal
- [ ] Non-fatal

30. Total number of fatal incidents reported *

31. Total number of injuries reported *

32. Total number of rescues reported *

33. Age range of cases *

34. Proportion of cases that were male *

35. Reported outcome measures (check all that apply) *

- [ ] Reported number/proportion of incidents
- [ ] Incidence/prevalence rates
- [ ] Risk/Odds ratios
- [ ] Univariate correlation among incidents
- [ ] multivariate correlation among incidents
- [ ] Other

36. Outcome data type (check all that apply) *

- ◯ Continuous outcome data
- ◯ Dichotomous outcome data

37. Key statistical findings (i.e., measures of risk, association - list all) *

38. Noted risk factors for exposure to environmental variable (i.e., high risk demographics, activities, other vulnerabilities) *

39. Study conclusions *

40. Limitations *

41. Climate change implications reported on *

This content is neither created nor endorsed by Microsoft. The data you submit will be sent to the form owner.

Microsoft Forms
